# Pathogen class-specific transcriptional responses derived from PBMCs accurately discriminate between fungal, bacterial, and viral infections

**DOI:** 10.1371/journal.pone.0311007

**Published:** 2024-12-12

**Authors:** Julie M. Steinbrink, Yiling Liu, Ricardo Henao, Ephraim L. Tsalik, Geoffrey S. Ginsburg, Elizabeth Ramsburg, Christopher W. Woods, Micah T. McClain

**Affiliations:** 1 Division of Infectious Diseases, Duke University, Durham, North Carolina, United States of America; 2 Computational Biology and Bioinformatics, Duke University, Durham, North Carolina, United States of America; 3 King Abdullah University of Science and Technology, Thuwal, Saudi Arabia; 4 Department of Biostatistics and Bioinformatics, Duke University, Durham, North Carolina, United States of America; 5 Danaher Diagnostics, United States of America; 6 Durham VA Health Care System, Durham, North Carolina, United States of America; 7 All of Us Research Program, National Institutes of Health, Bethesda, Maryland, United States of America; 8 Spark Therapeutics, Philadelphia, Pennsylvania, United States of America; University of Texas Medical Branch at Galveston, UNITED STATES OF AMERICA

## Abstract

Immune responses during acute infection often contain canonical elements which are shared across the responses to an array of agents within a given pathogen class (i.e., respiratory viral infection). Identification of these shared, canonical elements across similar infections offers the potential for impacting development of novel diagnostics and therapeutics. In this way, analysis of host gene expression patterns (‘signatures’) in white blood cells has been shown to be useful for determining the etiology of some acute viral and bacterial infections. In order to study conserved immune elements shared across the host response to related pathogens, we performed *in vitro* human PBMC challenges with common fungal pathogens (*Candida albicans*, *Cryptococcus neoformans* and *gattii*); four strains of influenza virus (Influenza A/Puerto Rico/08/34 [H1N1, PR8], A/Brisbane/59/2007 [H1N1], A/Solomon Islands/3/2006 [H1N1], and A/Wisconsin/67/2005 [H3N2]); and gram-negative (*Escherichia coli*) and gram-positive (*Streptococcus pneumoniae*) bacteria. Exposed human cells were then analyzed for differential gene expression utilizing Affymetrix microarrays. Analysis of pathogen exposure of PBMCs revealed strong, conserved gene expression patterns representing these canonical immune response elements to each broad pathogen class. A 41-gene multinomial signature was developed which correctly classified fungal, viral, or bacterial exposure with 94–98% accuracy. Furthermore, a 21-gene signature consisting of a subset of the discriminatory PBMC-derived genes was capable of accurately differentiating human patients with invasive candidiasis, acute viral infection, or bacterial infection (AUC 0.94, 0.83, and 0.96 respectively). These data reinforce the conserved nature of the genomic responses in human peripheral blood cells upon exposure to infectious agents and highlight the potential for *in vitro* models to augment our ability to develop novel diagnostic classifiers for acute infectious diseases, particularly devastating fungal infections.

## Introduction

Early differentiation of infected states remains critical to our ability to direct appropriate therapies and triage patients. This is particularly important in the setting of acute fungal infection, where delayed diagnosis can lead to delayed treatment, and thus significantly increased morbidity and mortality. The gold standard for diagnosis of acute infections has long revolved around targeted testing for specific pathogens or pathogen classes. Particularly, in fungal infections, this is often by biopsy with culture and histopathology review. However, such biopsies can often be difficult to obtain in the affected population due to the risks that come from such procedures, who frequently are also plagued by systemic immunosuppression and cytopenias [1]. Thus, additional diagnostics are clearly necessary.

There is a strong interest in utilizing patterns representing conserved elements of the host response to infectious stimulus to classify individuals as infected or noninfected, as well as to identify likely pathogen classes in order to better guide early therapy [2–7]. One of the prime targets of such research has been examination of host gene expression patterns in easily accessible peripheral blood. However, discovery of conserved peripheral blood genomic responses typically requires enrollment of a large number of individuals with the diseases in question as well as those with clinical mimics of the target disease. This requirement can be problematic due to the financial costs and time associated with enrolling clinical cohorts; as well as other logistical challenges including availability of cases/samples (as with rare infections, infections that are difficult to confirm as is seen with many invasive fungal pathogens, or diseases of less accessible global arenas) and danger to clinical and laboratory staff (as with hemorrhagic fever viruses and others). *In vitro* methods for screening for or validating such class-defining transcriptional signatures in circulating white blood cells, or peripheral blood mononuclear cells (PBMCs), offer the potential to mitigate many of these challenges [8–15].

In this paper, we demonstrate an *in vitro* method for identifying shared, canonical elements of the immune response to broad pathogen classes of clinical significance (fungal, bacterial, and viral) as well as establish its potential for application to real-world human cases. To do so we utilized a laboratory model where PBMCs were drawn from healthy individuals and then challenged *in vitro* with a variety of pathogens. Gene expression analyses were then performed on the human cells utilizing Affymetrix microarrays. Data was analyzed for patterns (or signatures) which were held in common amongst organisms of each class, followed by validation in cases of naturally acquired human infection.

## Methods

### *In vitro* stimulation of human PBMCs with various pathogens

Whole blood was drawn from six healthy individuals (3 male, 3 female age 25–35) and PBMCs were isolated via a standard Ficoll gradient procedure. Cells were then resuspended in RPMI 5 and plated in duplicate at a concentration of 6x10^6^ cells per well into 24-well plates. Relevant pathogens or controls were then added at different concentrations: *Candida albicans* SC 5314, *Cryptococcus neoformans* H99, and *Cryptococcus gattii* R265 at 10^6^ per well; Influenza viruses A/Wisconsin/67/2005 (H3N2), A/Brisbane/59/2007 (H1N1), A/PR8/34, and A/Solomon Islands/2007 (H1N1) at a final concentration of 10^3^ TCID_50_; and *Streptococcus pneumoniae* ATCC 6303 and *Escherichia coli* HST08 at 10^5^ per well; with each pathogen being used to infect cells from each of the six human donors. Fungi and bacteria were heat-killed prior to exposure to human cells to prevent overgrowth in culture medium. Cells were then incubated at 37 degrees with 5% CO2 for 24 hours, at which time cells were harvested and underwent centrifuge purification from culture media, as has been described previously [8, 9, 16]. Unexposed cells were harvested at 24 hours as controls.

### RNA extraction and microarray analysis

Cells were washed and placed in Qiagen RLT lysis buffer per manufacturer’s instructions and frozen for future gene expression analyses. At the time of gene expression analysis, RNA was then extracted (Qiagen, RNeasy Mini Extraction Kit, Germany), and hybridization and microarray data collection was performed at Expression Analysis (Durham, NC) using the GeneChip^®^ Human Genome U133A 2.0 Array (Affymetrix, Santa Clara, CA).

### Preprocessing of the gene expression data

Affymetrix data were preprocessed by using the gcrma R packages including optical noise and non-specific binding adjustments and quantile normalization [17]. Affymetrix probe IDs were mapped to gene symbols using Affymetrix Human Genome U133A 2.0 Array annotation data (chip hgu133a2) [18]. All analysis was performed in the R environment for statistical computing [19].

### Identification of differentially expressed genes and Gene Ontology enrichment analysis

The limma method was used to test for differential expression [20]. A mixed model was used to account for multiple samples from the same subject. P-values were considered significant if they achieved a Benjamini-Hochberg corrected false discovery rate (FDR) *p*-value of 0.05 [21]. Gene Ontology enrichment analysis was performed using the enrichR package [22, 23].

### Classification of perturbation classes

Sparse multinomial logistic regression was implemented by the R package glmnet to classify PBMC pathogen classes by taking the top 1500 probe sets with greatest variance (non-specific filtering) [24]. Least Absolute Shrinkage and Selection Operator (LASSO) regression was used to perform variable selection and regularization [25]. Predictions were generated using nested leave-one-out cross validation, and performance was assessed using the area under the receiving operating characteristic curve (AUC) and class-specific accuracies tabulated from a confusion matrix [26]. Signatures derived from the PBMC data were then validated in a dataset comprised of patients with acute infection due to the same broad pathogen classes (fungal, bacterial, viral) (S1 Table in S1 File). Signatures were validated by refitting a sparse multinomial regression using the PMBC-derived signature, as has been described [27].

## Results

### Experimental exposure of PBMCs to human pathogens results in marked changes at the transcriptomic level

For this experiment, we utilized a broad array of infectious pathogens including fungal stimuli with yeasts *Candida albicans*, *Cryptococcus neoformans*, and *Cryptococcus gattii;* four influenza virus strains; and bacterial stimuli with *Escherichia coli* and *Streptococcus pneumoniae*. These challenges were selected to represent a variety of major pathogen classes causing typical human infections.

**Fig 1:**
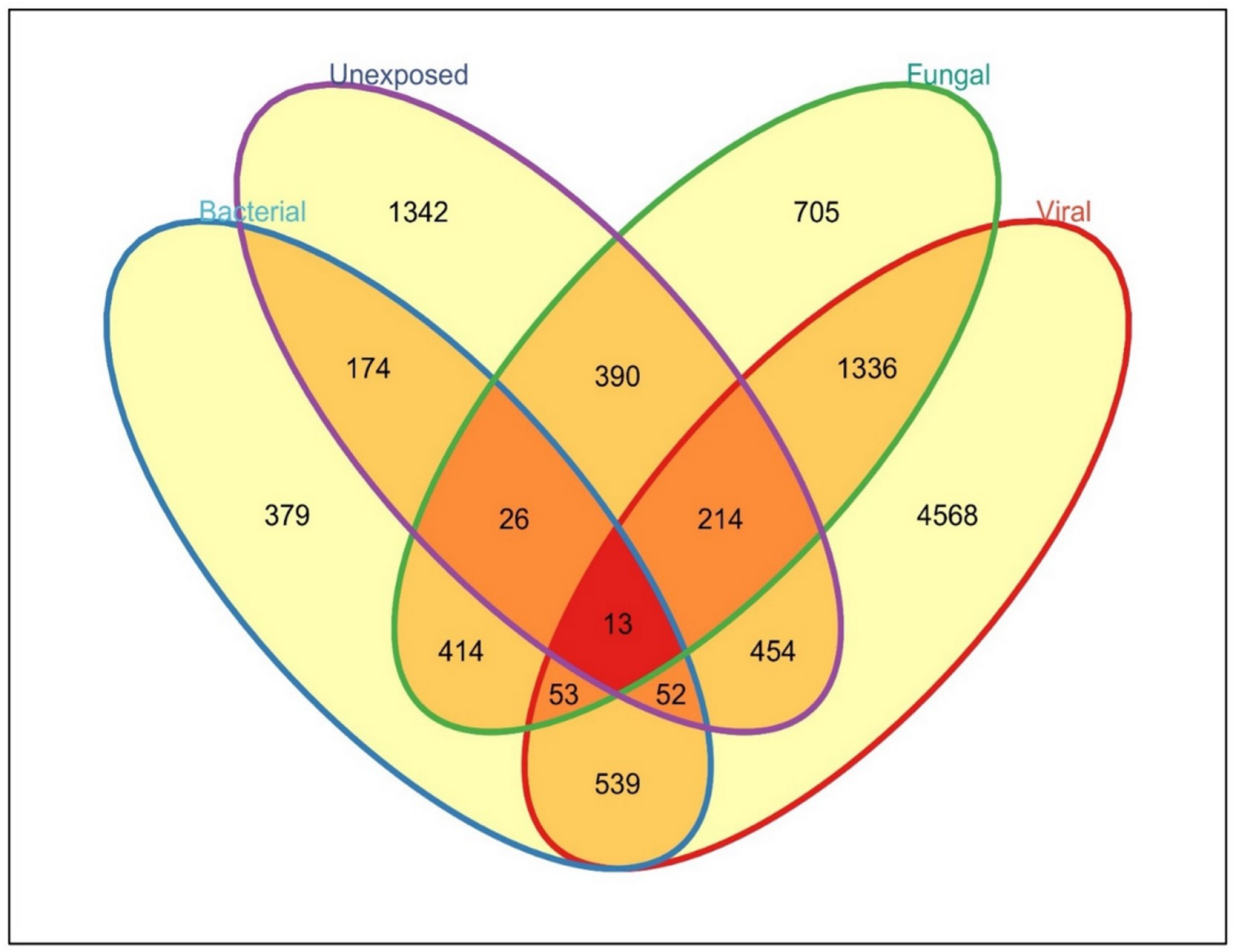
Unique and shared gene expression changes associated with exposure of human PBMCs to each pathogen class, or unexposed cells. Univariate comparisons between infection with each class of pathogens and all other groups are presented for each class.

We utilized univariate testing to determine sets of genes that exhibited differential expression between pathogen classes (each pathogen class vs. all other samples). At twenty-four hours post-exposure, the transcriptomic profiles of PBMCs exposed to each pathogen class were found to be robust and contained both overlapping as well as unique components (Fig 1). Influenza virus exposure resulted in the most marked changes in gene expression, with 3976 genes significantly upregulated and 592 genes downregulated compared to all other classes (4568 genes total). However, fungal exposure showed the next highest unique number of differentially regulated genes (705 total), followed by bacterial exposure (379 genes). Influenza exposure also demonstrated the greatest proportion of unique differentially expressed genes with 63% (4568/7229) of the influenza-associated genes differentially expressed only in viral exposure (vs all others), compared to 22% for both fungal (705/3151) and bacterial (379/1650) exposures. Fifty-three genes were upregulated in common amongst all exposure types compared to control cells, demonstrating that while there is a small generalized ‘pathogen exposure’ phenotype, the majority of the changes seen represent specific responses to individual pathogen classes.

### Functional annotation demonstrates that conserved biological pathways drive response to each pathogen

**Fig 2:**
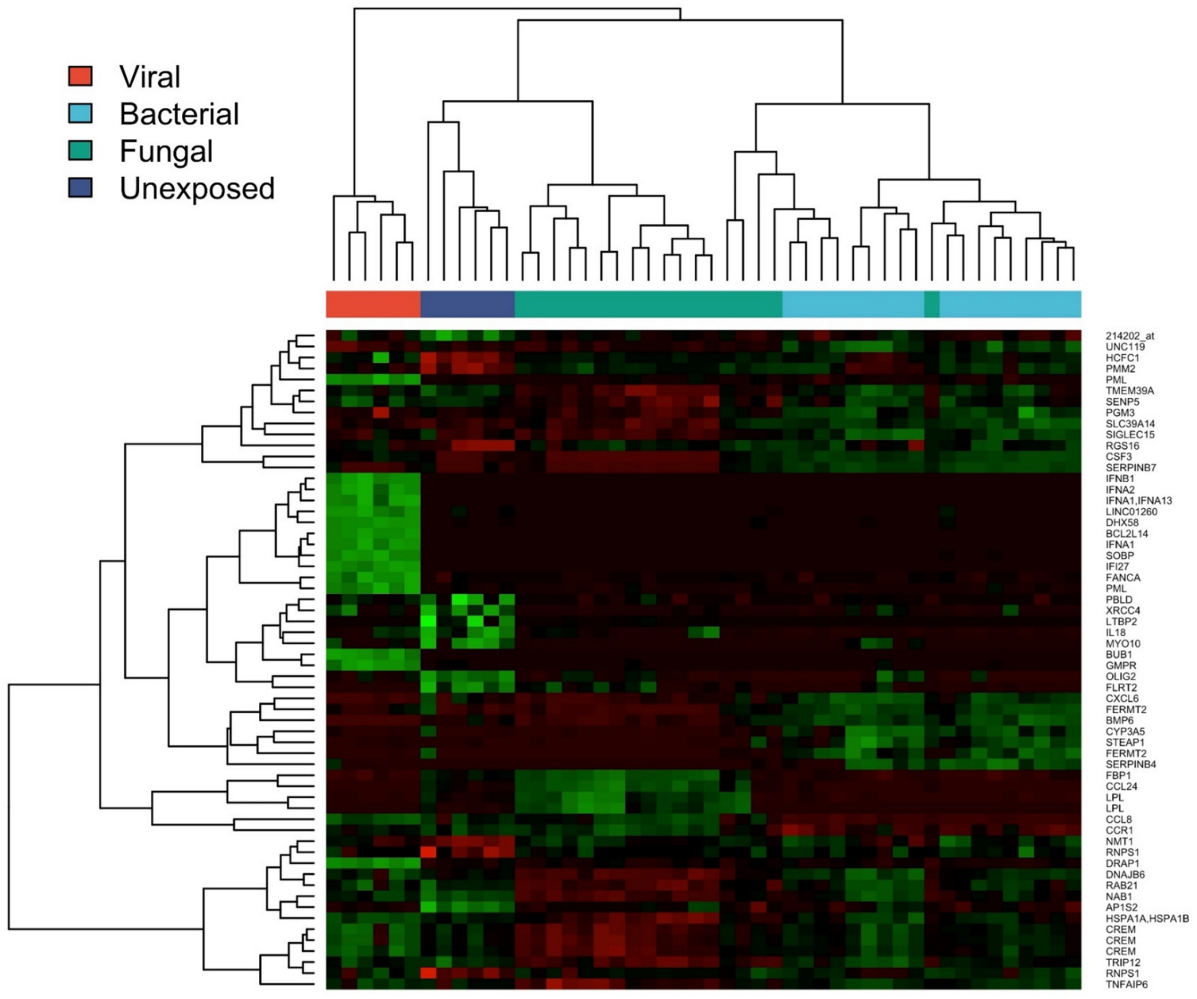
Differential gene expression in PBMCs exposed to various pathogen classes.

**Fig 3:**
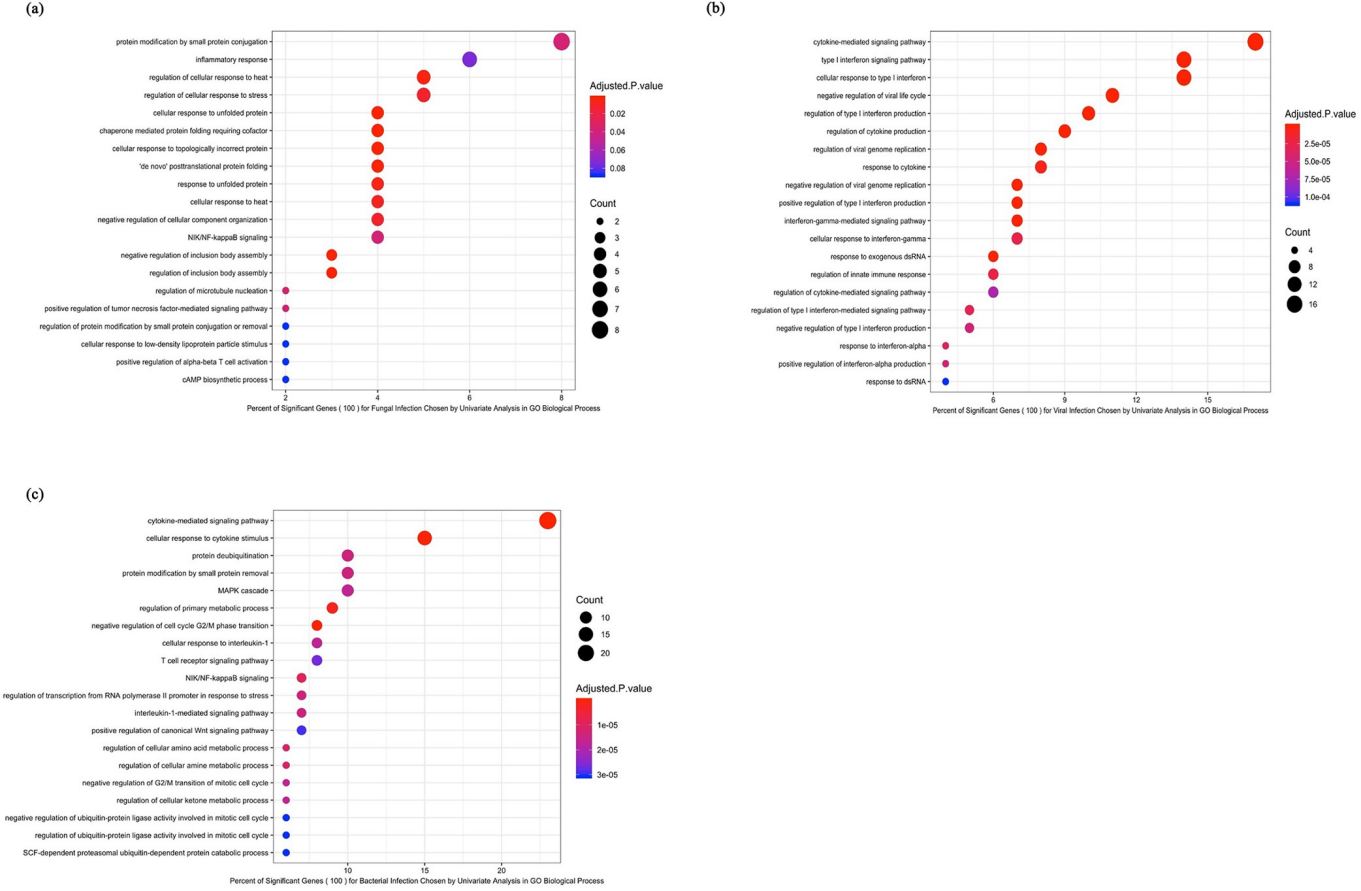
**A**. Biological pathways associated with transcriptomic responses to fungal stimulation of human PBMCs. **B.** Biological pathways associated with transcriptomic responses to viral stimulation of human PBMCs. **C.** Biological pathways associated with transcriptomic responses to bacterial stimulation of human PBMCs.

Analysis of individual genes as well as biological pathways represented by groups of genes revealed pathogen class-specific immune responses (Fig 2). Following exposure to the fungal pathogens *Candida* and *Cryptococcus*, a marked upregulation of inflammatory responses was seen including cellular responses to stress; eosinophil chemotaxis (CCL24, SOCS1/2); T cell proliferation and migration (IL2, CXCL9); and others (Fig 3A).

In the setting of viral exposure, the predominant biological pathways included those involved with cytokine signaling and interferon-responsiveness (Fig 3B). In cases of bacterial exposure, modular analysis of transcriptional responses demonstrated upregulation of genes known to play a role in both innate and acquired immunity, including non-interferon-based cytokine signaling, CCR1, CXCL6, CCL8, and the TNF receptor family (Fig 3C).

### PBMC-generated signatures have pathogen class specificity

**Fig 4:**
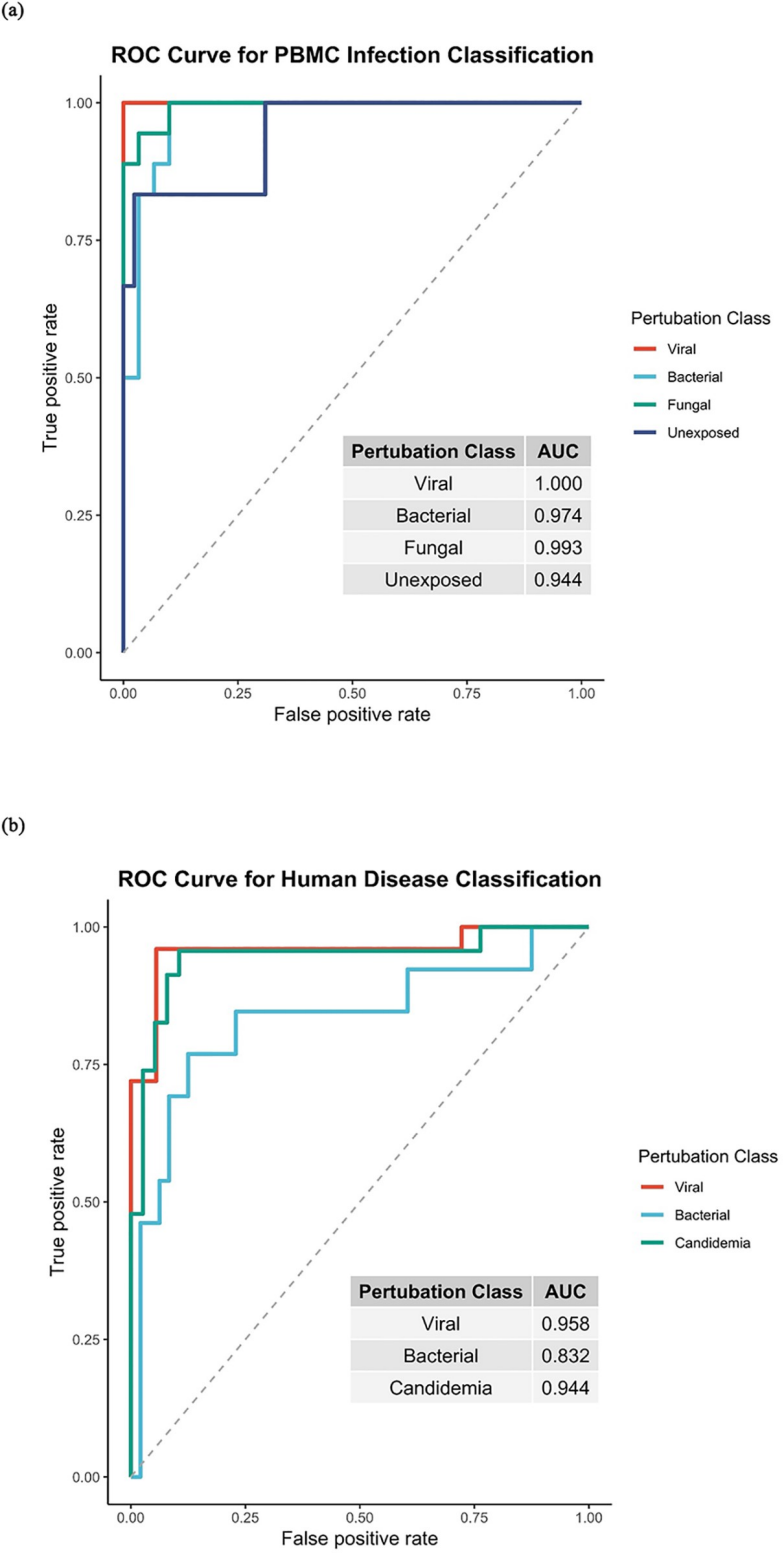
**A**. Performance of multinomial gene expression classifier distinguishing pathogen class. **B**. Validation of performance of the multinomial gene expression signature for diagnosis of acute infections in human patients. ROC = receiver operating curve; AUC = auROC, area under the receiver operating curve.

We next analyzed the data for patterns of gene expression (or signatures) that most accurately distinguished pathogen class. Using a sparse multinomial logistic regression model, we were able to generate a conserved 41-gene multinomial signature that offered a high degree of accuracy at diagnosing each type of exposure (S2 Table in S1 File). In this model, the fungal component of the signature (*Candida albicans*, *Cryptococcus neoformans*, and *Cryptococcus gattii* vs. all others) was capable of discriminating fungal challenge from bacterial and viral challenge and unexposed cells with a high degree of accuracy (AUC of 0.99, Fig 4A). The bacterial component of the signature performed almost as well at discriminating bacterial (*E*. *coli* and *S*. *pneumoniae* vs. all others) from viral and fungal challenge (AUC 0.97). The viral component of the signature had maximum performance, correctly classifying exposure with all influenza strains represented in the study compared to all other experimental conditions with 100% accuracy. However, no gene expression signatures were identified that could reliably differentiate between the four strains of influenza tested in this model (S1 Fig in S1 File).

### PBMC-generated gene signatures accurately classify natural human cases of disease

To validate whether the gene expression signatures obtained in this study could be used to correctly classify human subjects with acute febrile illness with similar accuracy, we applied the PBMC-derived signatures for each pathogen to a microarray dataset from peripheral blood samples of human patients with acute infection due to the same broad pathogen classes (S1 Table in S1 File). The banked blood samples from these subjects represented the time of initial presentation with fever from the indicated infection source [3, 28, 29]. We took the 41 genes from the PBMC classifier and repeated multinomial testing to re-derive a model with optimal sparsity and three possible outcomes (fungal, viral, or bacterial) utilizing microarray data from these human subjects (S3 Table in S1 File, Fig 4B). This resulted in a smaller, 21-gene classifier subset which performed with a high degree of accuracy (with leave-one-out cross-validation) in patients with acute infection (AUC for fungal, bacterial, and viral infection 0.94, 0.83, and 0.96 respectively) (Fig 4B, S2 Fig in S1 File).

## Discussion

Improvement in infectious diseases diagnostics is imperative to lead to earlier appropriate antimicrobials and improved clinical outcomes. Given the limitations of pathogen-based testing, an interest has developed in utilizing aspects of the host response for the development or validation of biomarkers to aid in pathogen diagnosis [2–7, 30, 31]. The investigation of this diagnostic approach is particularly challenging for fungal infections due to the difficulty identifying confirmed clinical cases compared to much more common bacterial and viral infections. As an alternative, *in vitro* exposures of human cells in culture have been used extensively for analyzing microbial pathophysiology and molecular and functional immune responses in host cells, and can therefore act as a model to evaluate the host gene expression response [10]. Herein we have demonstrated the ability of one such system to identify human gene expression signatures of fungal, viral, and bacterial exposure that not only discriminate pathogen class exposure *in vitro*, but also accurately classify natural cases of human infection in the real world.

The class-specific fungal, viral, and bacterial signatures defined herein exhibit some canonical immune response patterns [32–34]. This includes conserved gene expression pathways common to the host response to yeast pathogens (*Candida* and *Cryptococcus*), such as T cell signaling and eosinophil chemotaxis. This is similar to the stress-associated immune pathways seen by our group and others in the transcriptional responses of human subjects with candidemia, where responses reflected generalized cytokine signaling, inflammatory pathways, and cellular response to oxidative stress [35]. To further evaluate the biological plausibility of the PBMC-derived results in the setting of actual infection, we compared differential gene expression data from the fungal PBMC challenges to that seen in human patients with candidemia [35]. This demonstrated that 62% of the top 50 discriminatory genes from the PBMC challenge overlapped with responses seen in natural infection (S4 Table in S1 File) Twenty of these genes were upregulated, including those involved in immunoglobulin binding (FCGR1A and FCGR1B). Interestingly, downregulated genes also carried roles seen in systemic inflammatory response and susceptibility to infection, including TNSF12, CCR1, and CCR2.

In a murine model of invasive candidiasis, genes also clustered towards biological pathways reflective of generalized immune and defense response, as well as upregulation of inflammatory cytokines, including IL2, later in the disease course [36]. In an *in vitro* neutrophil model, “response to stress” was the most enriched Gene Ontology pathway after fungal exposure [37]. T cell responses similar to those found in our study have also been noted to play a significant role in the immune response to cryptococcal infections [38], along with innate and inflammatory immune responses seen in the transcriptional data of cryptococcal infection in a murine model [39], including inflammatory stress responses, chemokines involved in leukocyte attraction, and T cell signaling. Additionally, in our study, the top genes for fungal signature performance were enriched for chemokines involved in leukocyte attraction (including CCL2, CCL24, CCL7, CCL8, CCR1, CCR2) and immunoglobulin binding, again similar to transcriptional responses seen in murine models with invasive yeast infections [36, 39]. Whether these responses are conserved in cases of other pathogenic yeasts or molds remains to be fully clarified, though our prior work with a murine model of *Aspergillus* demonstrated similar results, including transcriptional responses reflective of cellular response to stress, inflammatory responses, cellular response to cytokine stimulus, and T cell activation and proliferation [40]. However, the high accuracy of a small gene expression signature to diagnose fungal infection with multiple different yeasts is encouraging in terms of its potential for broad clinical applicability. Even an initial distinction of fungal infection being present vs. not present, as opposed to a battery of individual fungal assays, has real-world implications in improving the initial triaging of patients and earlier appropriate antimicrobial therapy.

The genomic responses to *in vitro* challenge with *Candida* spp. have been previously examined in different ways [10, 11]. A study of the functional genomics of candidiasis (comparing *Candida*-stimulated PBMCs to unstimulated cells) showed some overlap in biologic pathways with our study (which focused on differentiating yeast infections from bacterial and viral disease), including lymphocyte proliferation and T cell differentiation, although not surprisingly only one individual gene overlapped between the top signatures in the two studies (CCL8) [11]. Another study utilized a different model (infection of whole blood, which includes neutrophils as well) and developed a signature representing genes whose expression differentiated *Candida* and *Aspergillus* spp. from bacterial stimulation [10]. Two genes overlapped between our yeast-derived classifier and the combined yeast/mold classifier from that model (SPRY2 and LPL). This variability demonstrated in ‘best’ genomic signatures through different laboratory and statistical models highlights the critical need for any such *in vitro*-derived signature to be validated in human cases of relevant disease. Encouragingly, we demonstrated in this study that our fungal findings can be translated to human patients with active *Candid*a infection. Over half of the top differentially expressed genes due to fungal exposure from our PBMC challenge overlapped with responses seen in patients with natural infection with candidemia. Furthermore, the yeast-associated host response genes with the best diagnostic performance *in vitro* also showed the ability to diagnose cases of invasive candidiasis in patients with a high degree of accuracy.

Despite being an *in vitro* model, PBMCs exposed to yeasts also demonstrate canonical responses thought to be critical to broad host defense mechanisms for fungal diseases, including Th17/IL-17 activation. *Candida albicans* is a direct inducer of anti-fungal Th17 and cross-reactive Th17 cells target *Candida* and other fungal species like *Aspergillus–*processes that lead directly to known human disease, as is seen in chronic mucocutaneous candidiasis [41]. These PBMCs also show evidence of activation of lipid and arachidonic acid metabolism pathways, other pathways known to be involved in susceptibility to *Candida* [42]. Additionally, yeast-infected PBMCs show marked upregulation of TLR4 and MCP-1/CCL2 as well as complement activation pathways, again mimicking innate responses that are critical for the antifungal response to *Candida* [11, 43, 44]. Taken together, these findings offer further support for the biological relevance of the model and its findings.

Similar to the findings following fungal exposure, bacterial stimulation also triggered highly canonical and specific antibacterial responses. The top genes for bacterial signature performance were enriched for interleukin signaling involving immunoregulation with both anti- (IL10, IL19) and pro- (IL1a, IL6, IL23) inflammatory components. The bacterial gene expression signature similarly showed significant overlap with published biological pathways of the host response to bacterial respiratory infection [3, 45–47]. This finding is consistent with the fact that circulating cells could have direct exposure to the bacterial pathogens tested (*S*. *pneumoniae* and *E*. *coli*) in the setting of bacteremia. The identification of a conserved signature that identifies both Gram-positive and Gram-negative infections is encouraging for the possible broad applicability of such a signature (similar to that described for the combined fungal signature), although additional testing with other pathogen types is needed to assess generalizability.

The top genes contributing to viral signature performance were enriched for interferon and interferon-response genes and included IFNA1, IFNA2, IFNB1, IFI44L, and IFI27, similar to prior published work [30, 48, 49]. When compared to gene expression data from acute respiratory viral infection in human patients [50], 84% of the top 50 discriminatory genes from the PBMC challenge overlapped with responses derived from human viral infections (S4 Table in S1 File) For the PBMC-generated signature of viral infection, we previously developed a signature of viral infection directly from human cases of influenza, and the transcriptional responses in both settings show a remarkable degree of overlap. This is of particular interest in the case of influenza infection, as while the cell types used to generate the two signatures are the same (total PBMCs), viremia and thus direct stimulation of circulating cells by virus is not thought to play a major role in the pathogenesis of most naturally-acquired infection [51]. Nonetheless, similar biological pathways seem to be stimulated in the PBMC populations in the two cases, enough so that the two signatures are practically interchangeable in terms of diagnostic accuracy. One hypothesis for this observed similarity is that many of the biological pathways that drive these signatures likely represent secondary signaling events. In the case of natural infection, these would be circulating leukocytes responding to chemokines from infected respiratory epithelia or resident macrophages, while in the *in vitro* model these may represent secondary responses to monocytes/macrophages that have internalized influenza antigens [52, 53]. Thus, in both cases the source of the strong gene expression signals detected are likely not productively infected cells *per se* but rather the larger body of circulating cells responding to cytokines and other mediators released from primary cells interacting with antigen [52].

This study does have several important limitations. First, a simple PBMC-based cell culture cannot mimic critically important interactions with non-circulating components of the host response to pathogen exposure including respiratory epithelium, tissue resident macrophages, lymph node activation, and others. Thus, significant components of the overall host response to a given exposure are not measured. Next, this study focused on a single post-exposure timepoint for analysis, and we do not know how *in vitro* timepoints compare to the various stages of natural human illness. It is possible that a larger study with dense serial sampling could uncover more accurate biomarker signatures across the full course of disease and would be more useful for defining the temporal biological breadth of the host response. While we have been able to develop gene expression signatures which are highly accurate for the specific pathogens tested (both *in vitro* and validated in actual cases of human infection), the generalizability of these pathogen-class signatures to additional untested pathogens within these classes (e.g., other gram positive bacteria or other *Candida* spp.) is unknown. Additionally, the bacterial and fungal stimuli used herein were heat-inactivated. Whether this had strong effects on critical structures involved in pathogen recognition is unclear, although the fact that we demonstrate similar gene expression responses in patients infected with similar wild-type pathogens argues that core elements of the innate responses to these heat-inactivated organisms are conserved. Lastly, we developed these biomarker signatures in cells from young, healthy PBMC donors. Future work will be required to assess how well these responses perform in cases where the host is subject to immunosuppression, as well as to compare how these signatures perform in patients across the age spectrum.

Many current diagnostic modalities require *a priori* a strong suspicion of the type of organism present in order to be effective–i.e., routine blood cultures will not pick up viral or many fungal infections, and traditional PCR and antigen testing require a specific known target. Novel methods of sequencing circulating nucleic acid to search for sequencing matches to known pathogen genomes offer the exciting potential for increased breadth of coverage agnostic to the clinical syndrome [54–56]. However, ongoing work remains to understand the sensitivity of such approaches as well as how to interpret the clinical relevance of positive results. As either an alternative or synergistic additional option, analysis of the host response to infection as manifested through gene expression in circulating cells offers the potential for an unbiased examination with pathogen class-specificity which has been lacking in most clinically available diagnostics. Furthermore, pauci-analyte biomarker panels based on gene targets like these lend themselves to conversion to point-of-care PCR platforms with the potential for sub-hour sample-to-answer times, significantly faster than most present fungal testing options. The ability for a single test to identify whether a primarily fungal, viral, or bacterial pathogen is driving an acute illness would help initiate appropriate class-specific therapies, and potentially reduce inappropriate overuse of antibacterials, such as in the case of fungal and viral infections [57]. Additionally, improved fungal diagnostics that lead to earlier appropriate antifungal initiation would result in improved clinical outcomes and decreased mortality in these critical diseases [58]. This investigative approach also has a potential role in the development of diagnostic signatures to emerging fungal pathogens and Biosafety Level (BSL) 3/4 pathogens for which human samples are rare or difficult to work with. While much work remains to be done, the data presented herein support the potential for pathogen class-specific host gene expression signatures to diagnose acute infection in both *in vitro* and human models.

## Conclusion

*In vitro* PBMC challenges with some pathogens trigger many of the canonical immune responses seen in circulating leukocytes during actual clinical illness in humans. These similarities to naturally-occurring immune responses permit such models to support the study of the pathophysiology of a wide array of infectious diseases as well as discovery of host transcriptomic signatures that can discriminate fungal, bacterial, and viral infection.

## Supporting information

S1 File Supplementary Figures and Tables, including: S1 Table: Top 50 Discriminatory Genes for Each Class vs All Others; S2 Table: Genes Involved in Each Phenotype of the PBMC Multinomial Signature; S3 Table: Genes Involved in Each Phenotype for the Multinomial Signature Applied to Human Subjects with Acute Infection; S1 Fig: Behavior of Canonical Antiviral Genes in Human PBMCs Stimulated Influenza and Uninfected Controls; S2 Fig: A 21-Gene Trinomial Classifier Contains Both Class-Unique and Overlapping Genes (A) and Differentiates Human Subjects with Acute Fungal (Candidemia), Viral, and Bacterial infection (B).
(DOCX)

## References

[1] Perfect JR. Fungal diagnosis: how do we do it and can we do better? Curr Med Res Opin. 2013;29 Suppl 4:3–11. Epub 2013/05/03. doi: 10.1185/03007995.2012.761134 . 23621588

[2] Bodkin N, Ross M, McClain MT, Ko ER, Woods CW, Ginsburg GS, et al. Systematic comparison of published host gene expression signatures for bacterial/viral discrimination. Genome Med. 2022;14(1):18. Epub 2022/02/22. doi: 10.1186/s13073-022-01025-x . 35184750 PMC8858657

[3] Tsalik EL, Henao R, Nichols M, Burke T, Ko ER, McClain MT, et al. Host gene expression classifiers diagnose acute respiratory illness etiology. Sci Transl Med. 2016;8(322):322ra11. doi: 10.1126/scitranslmed.aad6873 . 26791949 PMC4905578

[4] Herberg JA, Kaforou M, Wright VJ, Shailes H, Eleftherohorinou H, Hoggart CJ, et al. Diagnostic Test Accuracy of a 2-Transcript Host RNA Signature for Discriminating Bacterial vs Viral Infection in Febrile Children. JAMA. 2016;316(8):835–45. doi: 10.1001/jama.2016.11236 . 27552617 PMC5997174

[5] Liu X, Speranza E, Munoz-Fontela C, Haldenby S, Rickett NY, Garcia-Dorival I, et al. Transcriptomic signatures differentiate survival from fatal outcomes in humans infected with Ebola virus. Genome Biol. 2017;18(1):4. doi: 10.1186/s13059-016-1137-3 . 28100256 PMC5244546

[6] Chawla DG, Cappuccio A, Tamminga A, Sealfon SC, Zaslavsky E, Kleinstein SH. Benchmarking transcriptional host response signatures for infection diagnosis. Cell Syst. 2022;13(12):974–88.e7. Epub 2022/12/23. doi: 10.1016/j.cels.2022.11.007 . 36549274 PMC9768893

[7] Warsinske H, Vashisht R, Khatri P. Host-response-based gene signatures for tuberculosis diagnosis: A systematic comparison of 16 signatures. PLoS medicine. 2019;16(4):e1002786. Epub 2019/04/24. doi: 10.1371/journal.pmed.1002786 . 31013272 PMC6478271

[8] Das R, Hammamieh R, Neill R, Ludwig GV, Eker S, Lincoln P, et al. Early indicators of exposure to biological threat agents using host gene profiles in peripheral blood mononuclear cells. BMC Infect Dis. 2008;8:104. doi: 10.1186/1471-2334-8-104 . 18667072 PMC2542375

[9] Diaz-Mitoma F, Alvarez-Maya I, Dabrowski A, Jaffey J, Frost R, Aucoin S, et al. Transcriptional analysis of human peripheral blood mononuclear cells after influenza immunization. J Clin Virol. 2004;31(2):100–12. Epub 2004/09/15. doi: 10.1016/j.jcv.2004.04.006 . 15364265

[10] Dix A, Hunniger K, Weber M, Guthke R, Kurzai O, Linde J. Biomarker-based classification of bacterial and fungal whole-blood infections in a genome-wide expression study. Frontiers in microbiology. 2015;6:171. Epub 2015/03/31. doi: 10.3389/fmicb.2015.00171 . 25814982 PMC4356159

[11] Smeekens SP, Ng A, Kumar V, Johnson MD, Plantinga TS, van Diemen C, et al. Functional genomics identifies type I interferon pathway as central for host defense against Candida albicans. Nature communications. 2013;4:1342. Epub 2013/01/10. doi: 10.1038/ncomms2343 . 23299892 PMC3625375

[12] Zhang X, Mardinoglu A, Joosten LAB, Kuivenhoven JA, Li Y, Netea MG, et al. Identification of Discriminating Metabolic Pathways and Metabolites in Human PBMCs Stimulated by Various Pathogenic Agents. Front Physiol. 2018;9:139. Epub 2018/03/15. doi: 10.3389/fphys.2018.00139 . 29535640 PMC5835230

[13] Riege K, Holzer M, Klassert TE, Barth E, Brauer J, Collatz M, et al. Massive Effect on LncRNAs in Human Monocytes During Fungal and Bacterial Infections and in Response to Vitamins A and D. Sci Rep. 2017;7:40598. Epub 2017/01/18. doi: 10.1038/srep40598 . 28094339 PMC5240112

[14] Leonor Fernandes Saraiva JP, Zubiria-Barrera C, Klassert TE, Lautenbach MJ, Blaess M, Claus RA, et al. Combination of Classifiers Identifies Fungal-Specific Activation of Lysosome Genes in Human Monocytes. Frontiers in Microbiology. 2017;8(2366). doi: 10.3389/fmicb.2017.02366 29238336 PMC5712586

[15] Saraiva JP, Oswald M, Biering A, Röll D, Assmann C, Klassert T, et al. Fungal biomarker discovery by integration of classifiers. BMC Genomics. 2017;18(1):601. doi: 10.1186/s12864-017-4006-x 28797245 PMC5553868

[16] Le KTT, Chu X, Jaeger M, Plantinga JA, Matzaraki V, Withoff S, et al. Leukocyte-Released Mediators in Response to Both Bacterial and Fungal Infections Trigger IFN Pathways, Independent of IL-1 and TNF-α, in Endothelial Cells. Frontiers in Immunology. 2019;10. doi: 10.3389/fimmu.2019.02508 31708927 PMC6824321

[17] Wu J., Irizarry R., wcfJ.M.J.G. gcrma: Background Adjustment Using Sequence Information. R package version 2.52.0. (2018).

[18] Carlson M. hgu133a2.db: Affymetrix Human Genome U133A 2.0 Array annotation data (chip hgu133a2). R package version 3.2.3. (2016).

[19] R Core Team (2016) R: A Language and Environment for Statistical Computing. R Foundation for Statistical Computing, Vienna, Austria. https://www.R-project.org/.

[20] Ritchie ME, Phipson B, Wu D, Hu Y, Law CW, Shi W, et al. limma powers differential expression analyses for RNA-sequencing and microarray studies. Nucleic Acids Res. 2015;43(7):e47. Epub 2015/01/22. doi: 10.1093/nar/gkv007 . 25605792 PMC4402510

[21] Benjamini Y, Hochberg Y. Controlling the False Discovery Rate: A Practical and Powerful Approach to Multiple Testing. Journal of the Royal Statistical Society: Series B (Methodological). 1995;57(1):289–300. doi: 10.1111/j.2517-6161.1995.tb02031.x

[22] Chen EY, Tan CM, Kou Y, Duan Q, Wang Z, Meirelles GV, et al. Enrichr: interactive and collaborative HTML5 gene list enrichment analysis tool. BMC bioinformatics. 2013;14:128. Epub 2013/04/17. doi: 10.1186/1471-2105-14-128 . 23586463 PMC3637064

[23] Kuleshov MV, Jones MR, Rouillard AD, Fernandez NF, Duan Q, Wang Z, et al. Enrichr: a comprehensive gene set enrichment analysis web server 2016 update. Nucleic acids research. 2016;44(W1):W90–7. Epub 2016/05/05. doi: 10.1093/nar/gkw377 . 27141961 PMC4987924

[24] Friedman J, Hastie T, Tibshirani R. Regularization Paths for Generalized Linear Models via Coordinate Descent. Journal of statistical software. 2010;33(1):1–22. Epub 2010/09/03. doi: 10.1109/TPAMI.2005.127 . 20808728 PMC2929880

[25] Tibshirani R. Regression Shrinkage and Selection via the Lasso. Journal of the Royal Statistical Society Series B (Methodological). 1996;58(1):267–88.

[26] Fawcett T. An introduction to ROC analysis. Pattern Recognition Letters. 2006;27(8):861–74. doi: 10.1016/j.patrec.2005.10.010

[27] Mahle RE, Suchindran S, Henao R, Steinbrink JM, Burke TW, McClain MT, et al. Validation of a Host Gene Expression Test for Bacterial/Viral Discrimination in Immunocompromised Hosts. Clin Infect Dis. 2021;73(4):605–13. Epub 2021/01/20. doi: 10.1093/cid/ciab043 . 33462581 PMC8366815

[28] Tsalik EL, Li Y, Hudson LL, Chu VH, Himmel T, Limkakeng AT, et al. Potential Cost-effectiveness of Early Identification of Hospital-acquired Infection in Critically Ill Patients. Ann Am Thorac Soc. 2015. doi: 10.1513/AnnalsATS.201504-205OC . 26700878

[29] Tsalik EL, Langley RJ, Dinwiddie DL, Miller NA, Yoo B, van Velkinburgh JC, et al. An integrated transcriptome and expressed variant analysis of sepsis survival and death. Genome Med. 2014;6(11):111. Epub 2014/12/30. doi: 10.1186/s13073-014-0111-5 mc4274761. 25538794 PMC4274761

[30] McClain MT, Nicholson BP, Park LP, Liu TY, Hero AO 3rd, Tsalik EL, et al. A Genomic Signature of Influenza Infection Shows Potential for Presymptomatic Detection, Guiding Early Therapy, and Monitoring Clinical Responses. Open forum infectious diseases. 2016;3(1):ofw007. Epub 2016/03/05. doi: 10.1093/ofid/ofw007 . 26933666 PMC4771939

[31] Lukaszewski RA, Jones HE, Gersuk VH, Russell P, Simpson A, Brealey D, et al. Presymptomatic diagnosis of postoperative infection and sepsis using gene expression signatures. Intensive Care Med. 2022;48(9):1133–43. Epub 20220713. doi: 10.1007/s00134-022-06769-z . 35831640 PMC9281215

[32] Chen X, Liu S, Goraya MU, Maarouf M, Huang S, Chen JL. Host Immune Response to Influenza A Virus Infection. Frontiers in immunology. 2018;9:320. Epub 2018/03/21. doi: 10.3389/fimmu.2018.00320 . 29556226 PMC5845129

[33] Ramos-Sevillano E, Ercoli G, Brown JS. Mechanisms of Naturally Acquired Immunity to Streptococcus pneumoniae. Frontiers in immunology. 2019;10:358. Epub 2019/03/19. doi: 10.3389/fimmu.2019.00358 . 30881363 PMC6405633

[34] Duggan S, Leonhardt I, Hunniger K, Kurzai O. Host response to Candida albicans bloodstream infection and sepsis. Virulence. 2015;6(4):316–26. Epub 2015/03/19. doi: 10.4161/21505594.2014.988096 . 25785541 PMC4601378

[35] Steinbrink JM, Myers RA, Hua K, Johnson MD, Seidelman JL, Tsalik EL, et al. The host transcriptional response to Candidemia is dominated by neutrophil activation and heme biosynthesis and supports novel diagnostic approaches. Genome Med. 2021;13(1):108. Epub 2021/07/07. doi: 10.1186/s13073-021-00924-9 . 34225776 PMC8259367

[36] Zaas AK, Aziz H, Lucas J, Perfect JR, Ginsburg GS. Blood gene expression signatures predict invasive candidiasis. Science translational medicine. 2010;2(21):21ra17. Epub 2010/04/09. doi: 10.1126/scitranslmed.3000715 . 20374997

[37] Klassert TE, Hölzer M, Zubiria-Barrera C, Bethge J, Klaile E, Müller MM, et al. Differential Transcriptional Responses of Human Granulocytes to Fungal Infection with Candida albicans and Aspergillus fumigatus. Journal of Fungi. 2023;9(10):1014. doi: 10.3390/jof9101014 37888270 PMC10607568

[38] Gibson JF, Johnston SA. Immunity to Cryptococcus neoformans and C. gattii during cryptococcosis. Fungal genetics and biology: FG & B. 2015;78:76–86. Epub 2014/12/17. doi: 10.1016/j.fgb.2014.11.006 . 25498576 PMC4503824

[39] Holcomb ZE, Steinbrink JM, Zaas AK, Betancourt M, Tenor JL, Toffaletti DL, et al. Transcriptional Profiles Elucidate Differential Host Responses to Infection with Cryptococcus neoformans and Cryptococcus gattii. J Fungi (Basel). 2022;8(5). Epub 20220422. doi: 10.3390/jof8050430 . 35628686 PMC9143552

[40] Steinbrink JM, Zaas AK, Betancourt M, Modliszewski JL, Corcoran DL, McClain MT. A transcriptional signature accurately identifies Aspergillus Infection across healthy and immunosuppressed states. Transl Res. 2020;219:1–12. Epub 2020/03/14. doi: 10.1016/j.trsl.2020.02.005 . 32165060 PMC7170547

[41] Bacher P, Hohnstein T, Beerbaum E, Röcker M, Blango MG, Kaufmann S, et al. Human Anti-fungal Th17 Immunity and Pathology Rely on Cross-Reactivity against Candida albicans. Cell. 2019;176(6):1340–55.e15. Epub 2019/02/26. doi: 10.1016/j.cell.2019.01.041 . 30799037

[42] Jaeger M, Matzaraki V, Aguirre-Gamboa R, Gresnigt MS, Chu X, Johnson MD, et al. A Genome-Wide Functional Genomics Approach Identifies Susceptibility Pathways to Fungal Bloodstream Infection in Humans. J Infect Dis. 2019;220(5):862–72. doi: 10.1093/infdis/jiz206 . 31241743 PMC6667794

[43] de Vries DH, Matzaraki V, Bakker OB, Brugge H, Westra HJ, Netea MG, et al. Integrating GWAS with bulk and single-cell RNA-sequencing reveals a role for LY86 in the anti-Candida host response. PLoS Pathog. 2020;16(4):e1008408. Epub 20200406. doi: 10.1371/journal.ppat.1008408 . 32251450 PMC7173933

[44] Desai JV, Kumar D, Freiwald T, Chauss D, Johnson MD, Abers MS, et al. C5a-licensed phagocytes drive sterilizing immunity during systemic fungal infection. Cell. 2023;186(13):2802–22.e22. Epub 20230522. doi: 10.1016/j.cell.2023.04.031 . 37220746 PMC10330337

[45] Ramilo O, Allman W, Chung W, Mejias A, Ardura M, Glaser C, et al. Gene expression patterns in blood leukocytes discriminate patients with acute infections. Blood. 2007;109(5):2066–77. Epub 2006/11/16. doi: 10.1182/blood-2006-02-002477 . 17105821 PMC1801073

[46] Suarez NM, Bunsow E, Falsey AR, Walsh EE, Mejias A, Ramilo O. Superiority of transcriptional profiling over procalcitonin for distinguishing bacterial from viral lower respiratory tract infections in hospitalized adults. J Infect Dis. 2015;212(2):213–22. doi: 10.1093/infdis/jiv047 . 25637350 PMC4565998

[47] Wallihan RG, Suarez NM, Cohen DM, Marcon M, Moore-Clingenpeel M, Mejias A, et al. Molecular Distance to Health Transcriptional Score and Disease Severity in Children Hospitalized With Community-Acquired Pneumonia. Front Cell Infect Microbiol. 2018;8:382. Epub 2018/11/15. doi: 10.3389/fcimb.2018.00382 . 30425971 PMC6218690

[48] McClain MT, Henao R, Williams J, Nicholson B, Veldman T, Hudson L, et al. Differential evolution of peripheral cytokine levels in symptomatic and asymptomatic responses to experimental influenza virus challenge. Clin Exp Immunol. 2015. doi: 10.1111/cei.12736 . 26506932 PMC4750592

[49] Woods CW, McClain MT, Chen M, Zaas AK, Nicholson BP, Varkey J, et al. A Host Transcriptional Signature for Presymptomatic Detection of Infection in Humans Exposed to Influenza H1N1 or H3N2. PLoS One. 2013;8(1):e52198. Epub 2013/01/18. doi: 10.1371/journal.pone.0052198 . 23326326 PMC3541408

[50] Zaas AK, Chen M, Varkey J, Veldman T, Hero AO 3rd, Lucas J, et al. Gene expression signatures diagnose influenza and other symptomatic respiratory viral infections in humans. Cell Host Microbe. 2009;6(3):207–17. Epub 2009/08/12. doi: 10.1016/j.chom.2009.07.006 . 19664979 PMC2852511

[51] Tse H, To KKW, Wen X, Chen H, Chan K-H, Tsoi H-W, et al. Clinical and virological factors associated with viremia in pandemic influenza A/H1N1/2009 virus infection. PloS one. 2011;6(9):e22534–e. doi: 10.1371/journal.pone.0022534 . 21980333 PMC3181248

[52] Mock DJ, Frampton MW, Nichols JE, Domurat FM, Signs DJ, Roberts NJ Jr. Influenza Virus Infection of Human Lymphocytes Occurs in the Immune Cell Cluster of the Developing Antiviral Response. Viruses. 2018;10(8):420. doi: 10.3390/v10080420 . 30103427 PMC6115886

[53] Cline TD, Beck D, Bianchini E. Influenza virus replication in macrophages: balancing protection and pathogenesis. The Journal of general virology. 2017;98(10):2401–12. Epub 09/08. doi: 10.1099/jgv.0.000922 . 28884667 PMC5725990

[54] Schlaberg R, Chiu CY, Miller S, Procop GW, Weinstock G. Validation of Metagenomic Next-Generation Sequencing Tests for Universal Pathogen Detection. Arch Pathol Lab Med. 2017;141(6):776–86. Epub 2017/02/09. doi: 10.5858/arpa.2016-0539-RA . 28169558

[55] Graf EH, Simmon KE, Tardif KD, Hymas W, Flygare S, Eilbeck K, et al. Unbiased Detection of Respiratory Viruses by Use of RNA Sequencing-Based Metagenomics: a Systematic Comparison to a Commercial PCR Panel. J Clin Microbiol. 2016;54(4):1000–7. Epub 2016/01/29. doi: 10.1128/JCM.03060-15 . 26818672 PMC4809917

[56] Armstrong AE, Rossoff J, Hollemon D, Hong DK, Muller WJ, Chaudhury S. Cell-free DNA next-generation sequencing successfully detects infectious pathogens in pediatric oncology and hematopoietic stem cell transplant patients at risk for invasive fungal disease. Pediatr Blood Cancer. 2019;66(7):e27734. Epub 2019/04/04. doi: 10.1002/pbc.27734 . 30941906

[57] Trevas D, Caliendo AM, Hanson K, Levy J, Ginocchio CC, America ftIDSo. Diagnostic Tests Can Stem the Threat of Antimicrobial Resistance: Infectious Disease Professionals Can Help. Clinical Infectious Diseases. 2020;72(11):e893–e900. doi: 10.1093/cid/ciaa1527 33206946

[58] Taur Y, Cohen N, Dubnow S, Paskovaty A, Seo SK. Effect of antifungal therapy timing on mortality in cancer patients with candidemia. Antimicrobial agents and chemotherapy. 2010;54(1):184–90. Epub 2009/11/04. doi: 10.1128/AAC.00945-09 . 19884371 PMC2798557

