# Supplementary material for: Pathogen class-specific transcriptional responses derived from PBMCs accurately discriminate between fungal, bacterial, and viral infections
Source: PLoS One. 2024 Dec 12;19(12):e0311007. doi: 10.1371/journal.pone.0311007 (PMC11637350; doi:10.1371/journal.pone.0311007)
Supplement: S1 File — Supplementary Figures and Tables, including: S1 Table: Top 50 Discriminatory Genes for Each Class vs All Others; S2 Table: Genes Involved in Each Phenotype of the PBMC Multinomial Signature; S3 Table: Genes Involved in Each Phenotype for the Multinomial Signature Applied to Human Subjects with Acute Infection; S1 Fig: Behavior of Canonical Antiviral Genes in Human PBMCs Stimulated Influenza and Uninfected Controls; S2 Fig: A 21-Gene Trinomial Classifier Contains Both Class-Unique and Overlapping Genes (A) and Differentiates Human Subjects with Acute Fungal (Candidemia), Viral, and Bacterial infection (B). (DOCX) [file pone.0311007.s001.docx]

**Supplementary Figure 1.** Behavior of canonical antiviral genes in human PBMCs stimulated with 4 different strains of influenza (A/Puerto Rico/08/34 (“PR8”), A/Wisconsin/67/2005 (“WIS”), A/Solomon Islands/3/2006 (“Sol Is”), A/Brisbane/59/2007 (”BRIS”), and uninfected controls (‘Unstim’).


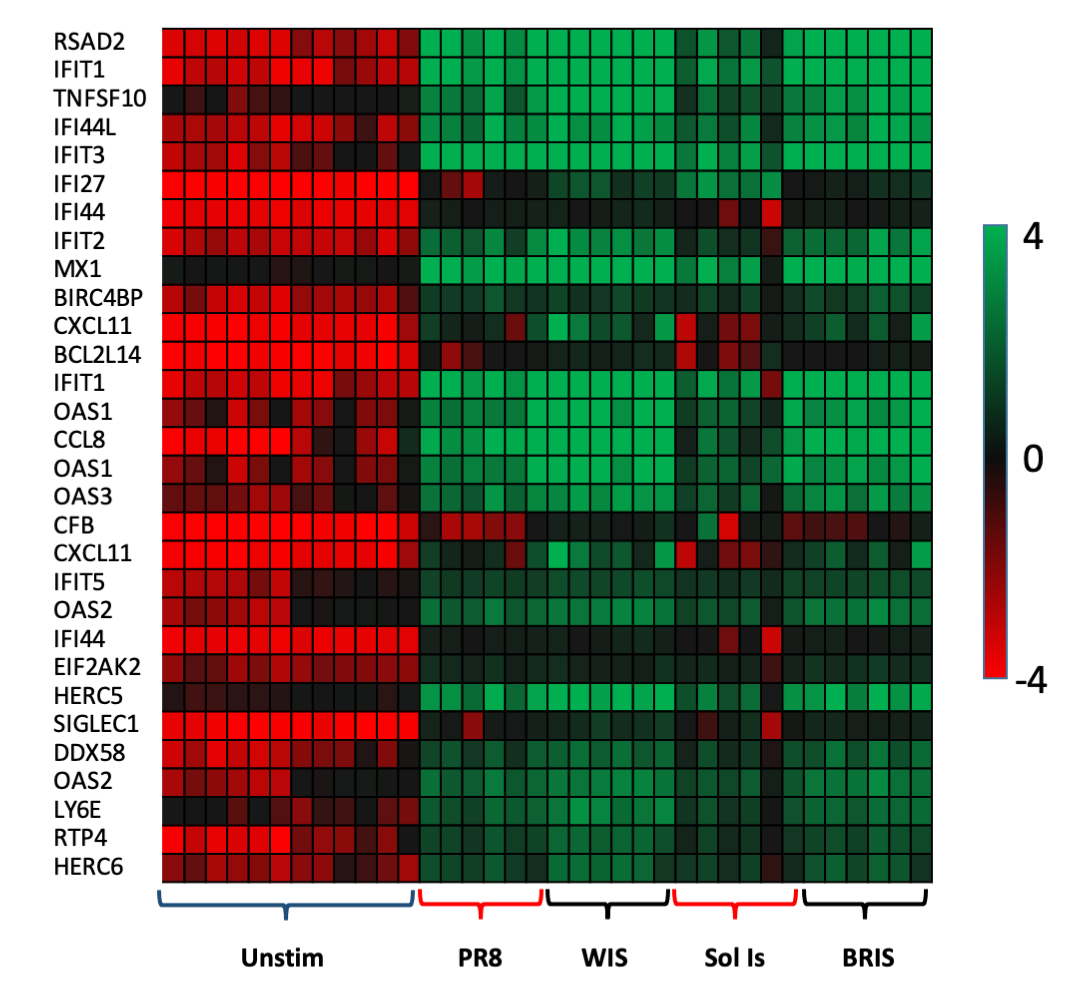


**Supplementary Figure 2.** A 21-gene trinomial classifier contains both class-unique and overlapping genes (A) and differentiates human subjects with acute Viral, Bacterial, and Fungal infection (Candidemia, panel B).


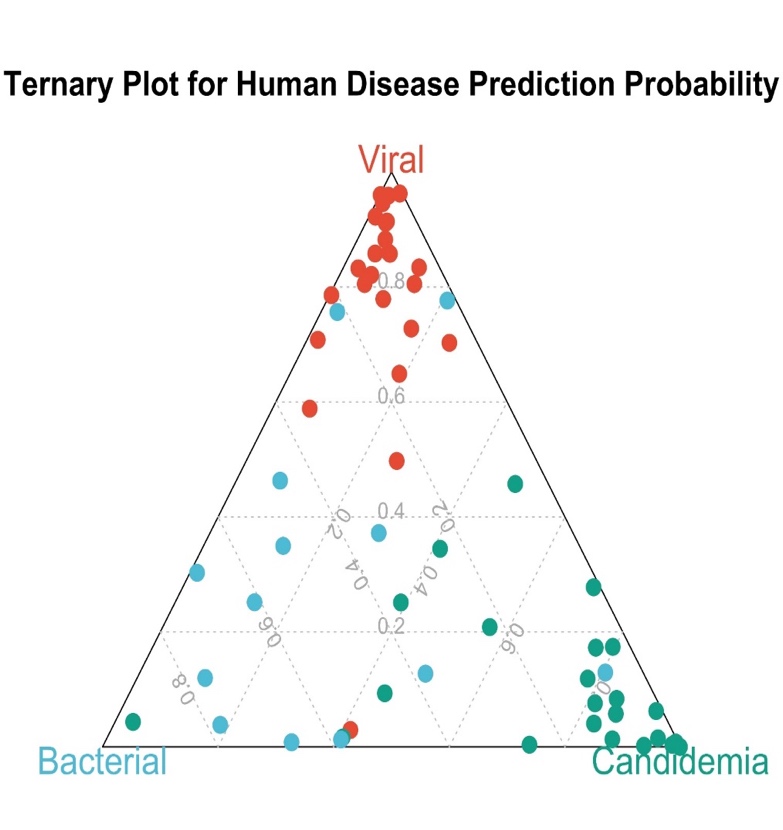

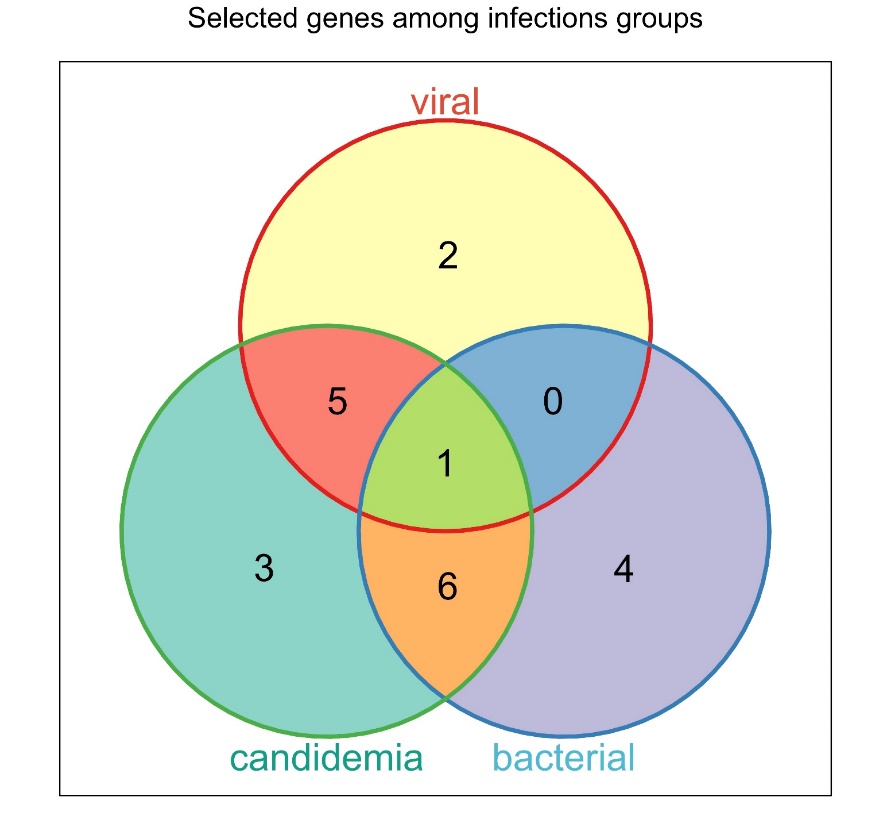


**A**

**B**

**B**

**Supplementary Material**

| **Supplementary Table 1. Dataset of Patients with Natural Infections** | | | | |
| --- | --- | --- | --- | --- |
| **Pathogen Class** | **Pathogen** | **Clinical Syndrome** | **Age** | **Sex** |
| Viral | Coronavirus NL63 | Respiratory infection | 67 | Male |
| Viral | Coronavirus NL63 | Respiratory infection | 64 | Male |
| Viral | Enterovirus/Rhinovirus | Respiratory infection | 65 | Male |
| Viral | Enterovirus/Rhinovirus | Respiratory infection | 68 | Male |
| Viral | Influenza A | Respiratory infection | 70 | Male |
| Viral | Influenza A | Respiratory infection | 64 | Male |
| Viral | Influenza A | Respiratory infection | 72 | Male |
| Viral | Influenza A | Respiratory infection | 69 | Male |
| Viral | Influenza A | Respiratory infection | 77 | Male |
| Viral | Influenza A (H3) | Respiratory infection | 89 | Male |
| Viral | Influenza A (H3) | Respiratory infection | 63 | Male |
| Viral | Influenza A (H3) | Respiratory infection | 66 | Male |
| Viral | Influenza A (H3) | Respiratory infection | 63 | Male |
| Viral | Influenza B | Respiratory infection | 66 | Male |
| Viral | Influenza B | Respiratory infection | 83 | Male |
| Viral | Influenza B | Respiratory infection | 60 | Male |
| Viral | Influenza B | Respiratory infection | 68 | Male |
| Viral | Influenza B | Respiratory infection | 60 | Male |
| Viral | Metapneumovirus | Respiratory infection | 60 | Male |
| Viral | Metapneumovirus | Respiratory infection | 62 | Male |
| Viral | Parainfluenza | Respiratory infection | 71 | Male |
| Viral | Respiratory Syncytial Virus | Respiratory infection | 67 | Male |
| Viral | Respiratory Syncytial Virus | Respiratory infection | 62 | Male |
| Viral | Respiratory Syncytial Virus | Respiratory infection | 72 | Male |
| Bacterial | Escherichia coli | Bacteremia | 69 | Female |
| Bacterial | Escherichia coli | Bacteremia | 81 | Female |
| Bacterial | Klebsiella pneumoniae | Bacteremia | 72 | Male |
| Bacterial | Escherichia coli | Urinary tract infection | 55 | Female |
| Bacterial | Escherichia coli | Bacteremia | 65 | Male |
| Bacterial | Escherichia coli | Urinary tract infection | 63 | Female |
| Bacterial | Staphylococcus aureus | Bacteremia | 74 | Male |
| Bacterial | Staphylococcus aureus | Bacteremia | 61 | Male |
| Bacterial | Methicillin Resistant Staphylococcus aureus | Bacteremia | 70 | Male |
| Fungal | Candida glabrata and Candida tropicalis | SOT, candidemia, empyema | 75 | Male |
| Fungal | Candida glabrata and Candida tropicalis | SOT, candidemia, empyema | 75 | Male |
| Fungal | Candida parapsilosis | SOT, candidemia, no metastatic sites | 45 | Female |
| Fungal | Candida parapsilosis | SOT, candidemia, no metastatic sites | 45 | Female |
| Fungal | Candida dubliniensis | Hematologic malignancy, candidemia, mediastinitis | 31 | Male |
| Fungal | Candida dubliniensis | Hematologic malignancy, candidemia, mediastinitis | 31 | Male |
| Fungal | Candida dubliniensis | Hematologic malignancy, candidemia, mediastinitis | 31 | Male |
| Fungal | Candida albicans | LVAD, candidemia, no metastatic sites | 34 | Male |
| Fungal | Candida albicans | LVAD, candidemia, no metastatic sites | 34 | Male |
| Fungal | Candida albicans | LVAD, candidemia, no metastatic sites | 34 | Male |
| Fungal | Candida albicans | Candidemia, no metastatic sites | 58 | Female |
| Fungal | Candida albicans | Candidemia, no metastatic sites | 58 | Female |
| Fungal | Candida glabrata | Candidemia, no metastatic sites | 66 | Male |
| Fungal | Candida glabrata | Candidemia, no metastatic sites | 62 | Male |
| Fungal | Candida glabrata | Candidemia, no metastatic sites | 62 | Male |
| Fungal | Candida albicans | Candidemia, no metastatic sites | 20 | Female |
| Fungal | Candida albicans | Candidemia, no metastatic sites | 20 | Female |
| Fungal | Candida parapsilosis | LVAD, candidemia, no metastatic sites | 50 | Female |
| Fungal | Candida parapsilosis | LVAD, candidemia, no metastatic sites | 50 | Female |
| Fungal | Candida parapsilosis | LVAD, candidemia, no metastatic sites | 50 | Female |
| Fungal | Candida glabrata | SOT, candidemia, no metastatic sites | 27 | Male |
| Fungal | Candida glabrata | SOT, candidemia, no metastatic sites | 27 | Male |
| Fungal | Candida albicans and Candida glabrata | Candidemia, no metastatic sites | 44 | Female |
| Fungal | Candida tropicalis | Candidemia, no metastatic sites | 57 | Female |
| Fungal | Candida albicans | LVAD, candidemia, no metastatic sites | 34 | Male |
| Fungal | Candida dubliniensis | Candidemia, no metastatic sites | 36 | Male |

**Supplementary Table 2. Genes Involved in Each Phenotype of the PBMC Multinomial Signature**

| **Bacterial** | **Fungal** | **Viral** | **Unexposed** |
| --- | --- | --- | --- |
| FERMT2 (6) | CYP27A1 (6) | IFI27 (5) | OLIG2 (6) |
| RGL1 (6) | IL17A1 (6) | BUB1 (5) | IL18 (8) |
| ENTPD1 (5) | LPL (5) | BCL2L14 (5) | PDGFC (4) |
| CCR1 (4) | UBFD1 (4) | SOBP (4) | RGS16 (4) |
| SERPIND1 (4) | SOCS2 (3) | IFNB1 (3) | TMEM45A (4) |
| SERPINB3, SERPINB4 (4) | SPRY2 (3) | IFNA2 (2) | TNS1 (3) |
| BMP6 (3) | TNFSF4 (3) |  | MYO10 (2) |
| CXCL6 (3) | TRIM36 (3) |  | FLRT2 (2) |
| SIGLEC15 (3) | GDF15 (3) |  | IL1RN (2) |
| ATF3 (2) | MAMLD1 (2) |  |  |
| SLC2A5 (2) |  |  |  |
| NMB (2) |  |  |  |
| GAGE12B, GAGE12F, GAGE12G, GAGE12I, GAGE4, GAGE5, GAGE6, GAGE7 (2) |  |  |  |
| KMO (2) |  |  |  |
| TNFRSF21 (2) |  |  |  |

*Counts in parentheses record how many times each gene was selected in LASSO with nested leave one subject out cross validation (# selected out of 6)

**Supplementary Table 3. Genes Involved in Each Phenotype for the Multinomial Signature Applied to Human Subjects with Acute Infection**

| **Bacterial** | **Candidemia** | **Viral** |
| --- | --- | --- |
| IFI27 (61) | SLC2A5 (61) | CYP27A1 (61) |
| ENTPD1 (61) | IL1RN (61) | CCR1 (61) |
| MYO10 (58) | PDGFC (60) | PDGFC (61) |
| TNFRSF21 (56) | MAMLD1 (59) | UBFD1 (59) |
| NMB (55) | GDF15 (59) | OLIG2 (8) |
| BMP6 (8) | ATF3 (19) | TNS1 (6) |
| AFT3 (5) | TNS1 (11) | TNFSF4 (1) |
| GDF15 (4) | UBFD1 (8) | IFNB1 (1) |
| SIGLEC15 (2) | CCR1 (6) |  |
| LPL (1) | IFI27 (5) |  |
| CYP27A1 (1) | MYO10 (2) |  |
|  | BMP6 (2) |  |
|  | LPL (1) |  |
|  | CYP27A1 (1) |  |
|  | OLIG2 (1) |  |

*Counts in parentheses record how many times each gene was selected in LASSO with nested leave one subject out cross validation (# selected out of 61).

**Supplementary Table 4. Top 50 Discriminatory Genes for Each Class vs All Others**

| Fungal | Viral | Bacterial |
| --- | --- | --- |
| CCL24 | CXCL11 | CSF3 |
| GPNMB | IFI27 | SERPINB7 |
| FBP1 | IFIT1 | IL36G |
| MRC1 | BCL2L14 | IL19 |
| MYOF | SOBP | CEMIP |
| CCL7 | USP18 | TM4SF1 |
| LPL | IFNB1 | SERPINB4 |
| ADORA2B | CD38 | MMP1 |
| CYP27A1 | IFNA2 | SLC22A1 |
| GDF15 | IFNA1 | IL24 |
| SPRY2 | CCL8 | CXCL13 |
| KLF4 | RTP4 | IL10 |
| CLEC5A | RSAD2 | CXCL6 |
| TGFBI | PGAP1 | PLA2G4A |
| FPR3 | CFB | STEAP1 |
| FCN1 | IFIT2 | BMP6 |
| SLAMF8 | CXCL10 | IL6 |
| CCR2 | OAS1 | PTGS2 |
| PLBD1 | SERPING1 | TNFRSF21 |
| CCL8 | IFI44L | ITGB8 |
| EGR2 | IFIT3 | SIGLEC15 |
| CD9 | IFI6 | F3 |
| MS4A4A | IFNG | FERMT2 |
| THBD | ETV7 | CSF2 |
| FCGR1B | TLR3 | SERPIND1 |
| TKTL1 | TLR7 | CYP27B1 |
| MITF | DEFB1 | IL1A |
| CYBB | HERC6 | EBI3 |
| FCGR1A | CYP2J2 | IL23A |
| TGM2 | OAS3 | PI3 |
| CYP1B1 | ZBP1 | ARNT2 |
| FOS | SPATS2L | TNIP3 |
| CD36 | MX1 | CD93 |
| PHLDA1 | DHX58 | CYP3A5 |
| CCL2 | SIGLEC1 | LIMK2 |
| CCR1 | IFI44 | CLGN |
| SPP1 | CXCL9 | TNFSF15 |
| LYZ | LAG3 | PLD1 |
| MERTK | CCL19 | SLCO4A1 |
| EIF1 | CASP10 | CA12 |
| TIMP2 | ISG15 | SERPINB3 |
| PLXDC2 | PRR5 | WNT5A |
| PLL1 | CFH | MET |
| APOC1 | ENPP2 | IL2RA |
| FCER2 | TNFSF10 | HBEGF |
| GREM1 | LY6E | CES1 |
| CSF1R | HERC5 | PTGES |
| PLPP3 | DDX58 | IL1RN |
| SOCS2 | IGFBP4 | CCL20 |
| TNFSF12 | IFNA10 | OSM |
